# Metabolomic Profiling Reveals the Quality Variations in *Citri Reticulatae* Pericarpium (*Citrus reticulata* Blanco cv. Chachiensis) with Different Storage Ages in Response to “*Candidatus* Liberibacter Asiaticus” Infection

**DOI:** 10.3390/foods13060827

**Published:** 2024-03-08

**Authors:** Jiayin Liang, Yuqing Xi, Jiaming Li, Shugui Xu, Yongqin Zheng, Meirong Xu, Zheng Zheng, Xiaoling Deng

**Affiliations:** 1 National Key Laboratory of Green Pesticide, South China Agricultural University, Guangzhou 510642, China; liangjiayin99@163.com (J.L.); 13928740803@163.com (Y.X.); lijiaming0216@stu.scau.edu.cn (J.L.); xushugui1126@foxmail.com (S.X.); zhengyongqin332@gmail.com (Y.Z.); meirongxu@scau.edu.cn (M.X.); 2 Guangdong Province Key Laboratory of Microbial Signals and Disease Control, South China Agricultural University, Guangzhou 510642, China

**Keywords:** *Citri Reticulatae* Pericarpium, *Citrus reticulata* blanco cv. chachiensis, huanglongbing, metabolites, quality variation

## Abstract

*Citri Reticulatae* Pericarpium, especially the pericarp of *Citrus reticulata* Blanco cv. Chachiensis (PCRC), is an important edible and medicinal ingredient for health and pharmacological properties. Citrus Huanglongbing, a devastating disease that currently threatens the citrus industry worldwide, is caused by a phloem-limited alpha-proteobacterium, “*Candidatus* Liberibacter asiaticus” (CLas). The industry of cultivar Chachiensis has been suffering from HLB. Although HLB affected the quality of citrus fruit, whether the quality of PCRC was affected by HLB remains unclear. In this study, we compared the metabolite profiles between HLB-affected and healthy PCRC from three sources: fresh, 6-month-old, and 9-year-old PCRC, through the untargeted LC–MS method. Compared to healthy controls, various types of bioactive compounds, mainly flavonoids, terpenoids, alkaloids, coumarins, polysaccharides, and phenolic acids, accumulated in HLB-affected PCRC, especially in the HLB-affected 9-year PCRC. In particular, isorhamnetin, isoliquiritigenin, luteolin 7-O-beta-D-glucoside, limonin, geniposide, pyrimidodiazepine, scoparone, chitobiose, m-coumaric acid, malonate, and pantothenic acid, which contributed to the pharmacological activity and health care effects of PCRC, were highly accumulated in HLB-affected 9-year-old PCRC compared to the healthy control. Multibioassay analyses revealed that HLB-affected 9-year-old PCRC had a higher content of total flavonoids and total polyphenols and exhibited similar antioxidant capacity as compared to healthy controls. The results of this study provided detailed information on the quality of HLB-affected PCRC.

## 1. Introduction

*Citri Reticulatae* Pericarpium (CRP), also known as “Chenpi” in Chinese, is the dried and aged pericarps of mandarin/tangerine (*Citrus reticulata* Blanco). CPR is known as an important source of nutraceuticals and functional food ingredients, as well as one of the most traditional Chinese herbal medicines used to treat digestive disorders or inflammatory respiratory diseases [1]. In China, CRP is also widely used as a culinary seasoning and dietary supplement [2]. Among CRPs, the pericarp of *C. reticulata* Blanco cv. Chachiensis (PCRC, “Guang Chenpi” in Chinese) is the most famous type, with excellent bioactive properties and various health benefits [3,4]. *C. reticulata* Blanco cv. Chachiensis was mainly grown in southern China. Particularly, plants cultivated in the Xinhui District and Jiangmen City of Guangdong Province were regarded as the most authentic ones. As a traditional Chinese herbal medicine, PCRP contains numerous bioactive compounds, including flavonoids, volatile oils, limonoids, phenolic acids, alkaloids, fatty acids, organic acids, polysaccharides, and amino acids [1]. Among these compounds, flavonoids and phenolic acids were two major metabolites in PCRP, which possessed various activities, including antioxidant, anti-inflammatory, anti-carcinogenic, and cardiovascular properties [4,5,6]. Previous studies have shown that the quality of PCRP is closely correlated with the storage time [7]. In particular, phenolic acids have been reported to be the main compounds that increase during the aging process of PCRP [7]. With its high medicinal and dietary value, the annual market sales of PCRC from Xinhui district have currently reached USD 145 million.

Citrus Huanglongbing (HLB), also known as citrus greening, is the most destructive disease threatening citrus production worldwide. HLB is caused by a phloem-limited alpha-proteobacterium, “*Candidatus* Liberibacter asiaticus” (CLas) [8]. Despite extensive research and efforts, there are still no effective strategies to control HLB or cure HLB-positive trees. Nearly all commercial citrus cultivars were susceptible to CLas [9,10]. In China, HLB was first reported in Guangdong Province around 1930 and caused large yield losses to citrus production in Guangdong Province after 1934 [11,12]. According to records, citrus exports were reduced by over 50% in the four main export cities of Guangdong province, including Shantou, Guangzhou, Jiangmen, and Gongbei, in 1934 [12]. Except for the excessive fruit drop, the quality of citrus fruit was also affected by HLB, including size, weight, flavor, and other variables such as acidity, Brix, and total soluble solids content [13,14].

Although *C. reticulata* Blanco cv. Chachiensis has been cultivated in the Xinhui District of Jiangmen City for hundreds of years, the production of *C. reticulata* Blanco cv. Chachiensis fruit and PCRP are still facing an unprecedented crisis due to the constant threat from HLB. HLB was first identified in *C. reticulata* Blanco cv. Chachiensis in Xinhui District around 1950, as reported by Lin Kong-Hsiang [11]. Since then, HLB has become an epidemic in the citrus-growing area of Xinhui District, destroying millions of citrus trees. The *C. reticulata* Blanco cv. Chachiensis industry in Xinhui District was nearly wiped out by HLB, with only 0.07 million mu of citrus planting area remaining by 1996, and it has gradually recovered since 2003. Currently, due to the high market demand, the price of *C. reticulata* Blanco cv. Chachiensis has reached USD 3 to 4.5 per kilogram for fresh fruit. In particular, the price of dried PCRP has exponentially increased with longer storage times. Therefore, due to the high value of PCRP and unmanageable HLB epidemics in Xinhui District, many local citrus growers are attempting to maintain the production of HLB-affected trees by applying additional fertilizers or using HLB-affected fruits for PCRP production. However, it is unclear whether HLB affects the quality of PCRC from HLB-affected fruit during storage.

In this study, HLB-affected and healthy PCRC from three sources, including fresh PCRC, 6-month PCRC, and 9-year PCRC, were collected. Untargeted liquid chromatography–mass spectrometry (LC–MS) was used to compare the metabolite profiles of healthy and HLB-affected PCRP. By comparing it with healthy PCRC, the global changes of components such as flavonoids, terpenoids, alkaloids, coumarins, amino acids, polysaccharides, fatty acids, phenolic acids, and organic acids in HLB-affected PCRP at different aging times were determined. The changes in the contents of total flavonoids (TPs), total polyphenols (TPs), and total antioxidant capacity (TAC) of HLB-affected and healthy PCRC were also determined using biochemical assays. The results of this study not only provided detailed information to demonstrate the quality of HLB-affected PCRC but also provided an essential scientific basis for using HLB-affected fruits as sources for PCRC production.

## 2. Materials and Methods

### 2.1. Sources of PCRC Samples

The fresh, healthy, and HLB-affected *C. reticulata* Blanco cv. Chachiensis fruits were collected from Xinhui District (22°11’60″ N, 113°0’0″ E) in Jiangmen City of Guangdong Province in September 2021. The pericarps of fresh fruits were immediately placed in liquid nitrogen and stored in a −80 °C freezer. The fresh pericarps of healthy fruits and HLB-affected fruits were naturally sun-dried for six months and stored in an airtight container for further analysis. The 9-year sun-dried PCRC of HLB-affected fruit originally collected from HLB-affected trees located in Xinhui District in September 2012 was selected as the candidate HLB-affected 9-year PCRC. As a control, the 9-year sun-dried PCRC of healthy fruit collected from the same region of Xinhui District was selected. The fruit pith (fresh PCRC) or endocarp (6-month and 9-year PCRC) tissue of each healthy and HLB-affected PCRC was sampled and used for DNA extraction and CLas detection. Based on the detection results, fresh PCRC (2021), 6-month dried PCRC (2021), and 9-year dried PCRC (2012) from healthy and HLB-affected fruits were selected for untargeted LC–MS analyses and multibioassay analyses.

### 2.2. DNA Extraction and CLas Detection

For fresh PCRC samples, 50 mg of fruit pith tissue was collected for DNA extraction. For dried PCRC samples, 50 mg of endocarp tissue was collected for DNA extraction. All tissue was cut into sections approximately 1 mm wide and crushed using an MP FastPrep 24 Grinder (MP Biomedicals LLC, Santa Ana, CA, USA) at a speed of 4 M/S for 2 min. Total DNA was extracted using the E.Z.N.A. HP Plant DNA Kit (OMEGA BioTek Co., Guangdong, China) following the manufacturer’s manual. The quality and quantity of DNA extracts were assessed spectrophotometrically using NanoDrop One (Thermo Fisher Scientific Inc., Singapore). CLas detection was performed using SYBR Green Real-time PCR with two sets of primers, CLas4G/HLBr and RNRf/RNRr, according to previous studies [15,16]. The specific primer (18S-F/18S-R) sets targeting the citrus 18S rRNA genes were selected as a reference for citrus [17]. All primers used in this study are listed in Table S1. All SYBR Green Real-time PCR was performed in the CFX Connect Bio-Rad Real-time PCR System (Bio-Rad Laboratories Inc., Hercules, CA, USA). The PCR mixture (20 μL) contained 10 μL of TransStart^®^ Green qPCR SuperMix (TransGen Biotech, Beijing, China), 0.4 μL of each forward and reverse primer (10 μM), 1 μL of DNA template, and 8 μL of ddH_2_O. The PCR procedure included incubation at 95 °C for 30 s followed by 40 cycles of amplification (95 °C for 10 s and 58 °C for 30 s) with fluorescence signal capture at the end of each 60 °C step. The result (Ct value) of Real-time PCR was analyzed using Bio-Rad CFX Manager 2.1 software with automated baseline settings and thresholds. To screen for more credible HLB-affected pericarp samples, only DNA samples with a Ct value <30 were considered HLB-affected samples and used for further analyses.

### 2.3. Untargeted Metabolomics Analysis

Each sample was added with 600 μL MeOH (Fisher Scientific, Loughborough, UK) containing 2-Amino-3-(2-chloro-phenyl)-propionic acid (4 ppm) (Aladdin, Shanghai, China) into a tissue grinder. After grinding at 60 Hz for 90 s, the samples were treated with ultrasound for 15 min and then centrifuged at 12,000 rpm for 10 min at 4 °C. The supernatant was separated by filtration through a 0.22 µm membrane. The filtrate was collected and added to the detection bottle for LC–MS analyses.

The LC–MS analysis was performed on a Vanquish UHPLC System (Thermo Fisher Scientific, USA). Chromatography analysis was carried out with an ACQUITY UPLC^®^ HSS T3 (150 × 2.1 mm, 1.8 µm) (Waters, Milford, MA, USA). For LC–ESI (-)-MS analysis, the analytes were carried out with (A) acetonitrile and (B) ammonium formate (5 mM) and LC–ESI (+)-MS analysis; (C) the mobile phases consisted of 0.1% formic acid in acetonitrile (*v*/*v*) and (D) 0.1% formic acid in water (*v*/*v*), respectively. The separation of components from PCRC was conducted under the following gradient elution: 0~1 min, 2% A/C; 1~9 min, 2~50% A/C; 9~12 min, 50~98% A/C; 12~13.5 min, 98% A/C; 13.5~14 min, 98~2% A/C; 14~20 min, 2% A/C. The flow rate and injection volume were set at 0.25 mL/min and 2 μL, respectively. Mass spectrometric detection of metabolites was performed on the Orbitrap Exploris 120 (Thermo Fisher Scientific, Waltham, MA, USA) with an ESI ion source. Simultaneous MS1 and MS/MS (full MS-ddMS2 mode, data-dependent MS/MS) acquisition was applied. MS1 resolving power was set at 60,000 FWHM, and MS/MS resolving power was set at 15,000 FWHM. Data collection was performed through a series of energy collisions, automatically eliminating useless information.

### 2.4. Data Processing and Chemical Metabolite Identification

The original mass spectrometer off-camera file was normalized into mzXML file format by using the MSConvert tool from the Proteowizard software package (v3.0.8789) [18]. Substances were further identified using the public databases HMDB (http://www.hmdb.ca (accessed on 27 May 2022)), massbank (http://www.massbank.jp/ (accessed on 27 May 2022)), LipidMaps (http://www.lipidmaps.org (accessed on 27 May 2022)), mzcloud (https://www.mzcloud.org (accessed on 27 May 2022)), KEGG (http://www.genome.jp/kegg/ (accessed on 27 May 2022)) and self-built substance library by setting ppm < 30. Data correction and elimination of systematic errors in all PCRC samples were performed with the LOESS signal correction method based on QC samples [19]. Substances with an RSD > 30% in the QC samples were filtered out of the data quality control. A principal component analysis (PCA) was applied to reduce dimensionality and visualize the main correlations in the data under an unsupervised method. The supervised partial least squares-discriminant analysis (PLS-DA) was used to discriminate and identify potential key biomarkers among samples (http://v2.biodeep.cn/tools (accessed on 27 May 2022)). The OPLS-DA dimensionality reduction method was used to calculate the variable projection importance (VIP). The strength of influence and interpretation ability of each metabolite component’s content on the classification and discrimination of samples were measured to assist in the screening of marker metabolites. Metabolite molecules were considered statistically significant between HLB-affected PCRC and healthy PCRC when the |Log2 fold change| > 1, *p*-value value < 0.05, and VIP > 1.

### 2.5. Determination of Total Flavonoids (TFs) Content, Total Polyphenols (TPs) Content, and Total Antioxidant Capacity (TAC) of PCRC

For each type of PCRC, the same samples used for LC–MS analyses were selected for multibioassay analyses. Each PCRC sample was initially added to 1 mL of 60% ethanol for extraction for 30 min, and then centrifuged at 12,000 rpm for 10 min. The supernatant was taken and fixed to 1 mL with 60% ethanol for the determination of TF and TP content. 

The TF content was determined using the Plant Flavonoids Content Assay Kit (Beijing Solarbio Science & Technology Co., Ltd., Beijing, China) according to the instructions. Rutin was used as a standard and diluted into eight gradients (1.50000 mg/mL, 1.25000 mg/mL, 0.62500 mg/mL, 0.31250 mg/mL, 0.15625 mg/mL, 0.07800 mg/mL, 0.03900 mg/mL, and 0.02000 mg/mL). Absorbance was measured at 470 nm using a multifunctional microplate reader (Synergy H1, BioTek, USA). The TF content of each sample was indicated as mg of rutin equivalents per g dry weight of PCRC (mg/g d.w.). 

The TP content was determined by the Folin–Ciocalteu colorimetric method using the Plant Total Phenol Content Assay Kit (Beijing Solarbio Science & Technology Co., Ltd.) according to the instructions. Gallic acid was used as a standard and diluted into eight gradients (0.625000 mg/mL, 0.156250 mg/mL, 0.078125 mg/mL, 0.039000 mg/mL, 0.020000 mg/mL, 0.01 mg/mL, 0.005000 mg/mL, and 0.002500 mg/mL). Absorbance was measured at 760 nm using a multifunctional microplate reader (Synergy H1, BioTek, Winooski, VT, USA). The TF content of each sample was indicated as mg of gallic acid equivalents per g dry weight of PCRC (mg/g d.w.). 

The TAC of each PCRC sample was determined by the ferric-reducing antioxidant power (FRAP) method using the Total Antioxidant Capacity Assay Kit (Beijing Solarbio Science & Technology Co., Ltd.). Absorbance was measured at 593 nm using a multifunctional microplate reader (Synergy H1, BioTek, Winooski, VT, USA). The FRAP value of samples was calculated from the linear calibration curve and expressed as μmol of Fe^2+^ equivalents per g dry weight of PCRC (μmol/g d.w.).

### 2.6. Statistical Analysis

All experiments were performed in three biological replications. Statistical analysis was conducted using SPSS v25.0 software. A one-way analysis of variance (ANOVA) test (based on Duncan’s test at *p* < 0.05) and correlation analysis (Pearson) were used to analyze statistically significant differences between HLB-affected PCRC and healthy PCRC groups. Graphs were edited with Origin 2021 software.

## 3. Results

### 3.1. CLas Detection and Phenotypic Characteristics of PCRC Samples

Quantification results showed that all HLB-affected fresh PCRC and HLB-affected 6-month PCRC were CLas-positive with a higher concentration based on primer sets CLas4G/HLBr (Ct values ranging from 15.58 to 24.19) and RNRf/RNRr (Ct values ranging from 14.96 to 23.42) (Table S2). However, among 28 candidate HLB-affected 9-year PCRCs, five (17.8%) showed Clas-positive with a moderate concentration, with a Ct value ranging from 24.21 to 28.27 (Table S2). A significantly higher Ct value of primer 18S-F/18S-R was observed in both healthy and candidate HLB-affected 9-year PCRC DNA as compared to the fresh PCRC and 6-month PCRC DNA samples (Table S2), indicating stronger DNA degradation in long-term stored PCRC. In addition, all fresh pericarps, 6-month pericarps, and 9-year pericarps from healthy fruit showed CLas negative (Table S2). 

**Figure 1:**
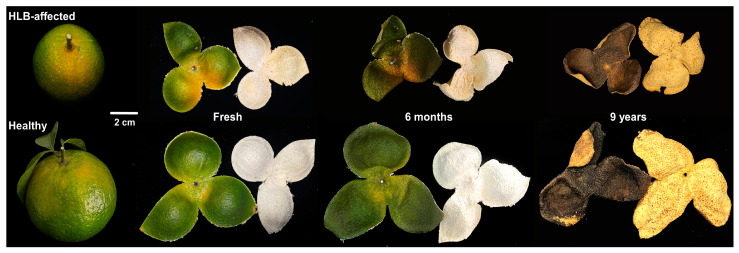
Images of healthy and HLB-affected fruit and pericarp of *Citrus reticulata* Blanco cv. Chachiensis (PCRC). Fresh: fresh PCRC; 6 months: sun-dried PCRC that was stored for six months; 9 years: sun-dried PCRC that was stored for nine years.

Compared to the healthy fresh PCRC, the HLB-affected fresh PCRC showed uneven coloring, i.e., the color of the pericarp at the stem end changed from green to yellow, and other parts of the pericarp remained green (Figure 1). The size of HLB-affected PCRCs from three sources was smaller than that observed in the corresponding healthy PCRC (Figure 1). It was also found that HLB-affected 9-year PCRC exhibited lighter color and thinner thickness as compared to the healthy 9-year PCRC (Figure 1).

### 3.2. Multivariate Analyses of Metabolites in Healthy and HLB-Affected PCRC

**Figure 2:**
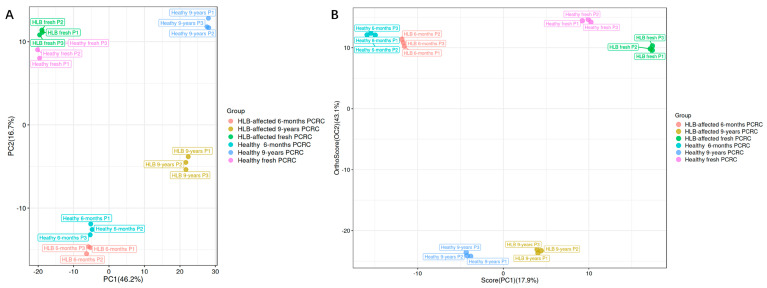
The PCA scatter plot (**A**) and the OPLS−DA score plot (**B**) of healthy and Huanglongbing (HLB)−affected pericarp of *Citrus reticulata* Blanco cv. Chachiensis (PCRC) from three sources (fresh, 6 months, and 9 years).

The PCA scatter plot showed that PC1 and PC2 explained 62.9% of the total difference, with PC1 accounting for 46.2% and PC2 accounting for 16.7% of the total variation in normalized LC–MS data (Figure 2A). The supervised OPLS-DA mode was also adopted to show the compound differences between groups. In the OPLS-DA score plot, three replicate samples from the same group were more closely clustered (Figure 2B), displaying good repeatability of the untargeted LC–MS method. Four separate clusters were observed, including the fresh PCRC group, the 6-month PCRC group, the healthy 9-year PCRC group, and the HLB-affected 9-year PCRC group (Figure 2B). For the fresh and 6-month PCRC group, the healthy and HLB-affected PCRC were closely clustered (Figure 2B). However, within the 9-year PCRC group, the healthy PCRC and HLB-affected PCRC were separately situated at two sides of the PC2 axis (Figure 2B). This suggested that the global metabolites between long-term stored healthy and HLB-affected PCRC were much greater than those observed between short-term stored healthy and HLB-affected PCRC. In addition, the heatmap of metabolites showed the same cluster result as PCA among different types of PCRC (Figure S1).

### 3.3. Comparative Metabolic Analysis of Healthy and HLB-Affected PCRC

**Figure 3:**
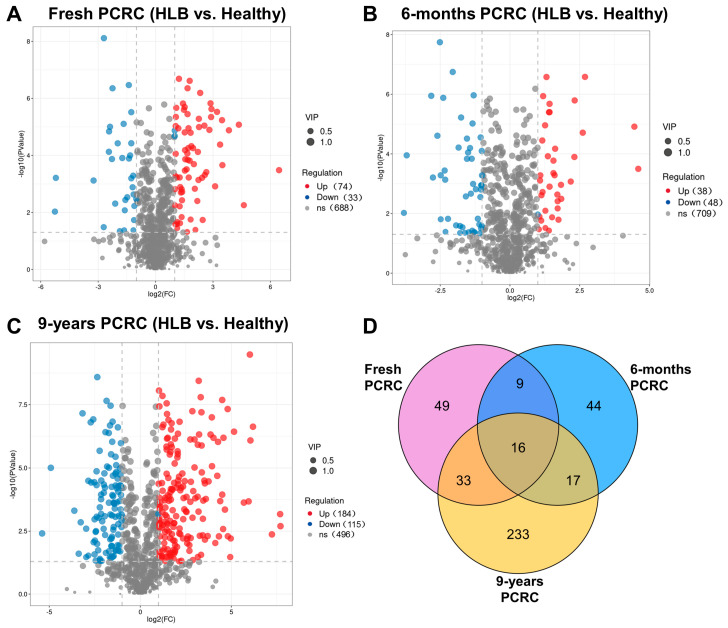
Volcano plot and Venn diagram of differential metabolites between healthy and Huanglongbing (HLB)−affected pericarp of *Citrus reticulata* Blanco cv. Chachiensis (PCRC). (**A**) HLB-affected fresh PCRC vs. healthy fresh PCRC. (**B**) HLB−affected 6−month PCRC vs. healthy 6−month PCRC. (**C**) HLB−affected 9−year PCRC vs. healthy 9−year PCRC. (**D**) Venn cluster of differential metabolites among three groups.

Compared to the healthy fresh PCRC, 107 differentially abundant metabolites were identified in the HLB-affected fresh PCRC (*p* < 0.05, VIP > 1), including 74 up-regulated and 33 down-regulated (Figure 3A). A total of 86 differentially abundant metabolites (*p* < 0.05, VIP > 1) were identified in the methanol extraction of HLB-affected 6-month PCRC compared to the healthy control, of which 38 were up-regulated and 48 were down-regulated (Figure 3B). In HLB-affected 9-year PCRC, 299 metabolites showed differential abundance as compared to healthy 9-year PCRC (*p* < 0.05, VIP > 1), including 184 up-regulated and 115 down-regulated metabolites (Figure 3C). The Veen cluster identified 16 common metabolites that were differentially abundant among the three groups (Figure 3D). A total of 233 altered metabolites were uniquely found in HLB-affected 9-year PCRC, while 49 and 44 altered metabolites were identified in HLB-affected fresh PCRC and HLB-affected 6-month PCRC, respectively (Figure 3D).

### 3.4. KEGG Pathway Analysis

**Figure 4:**
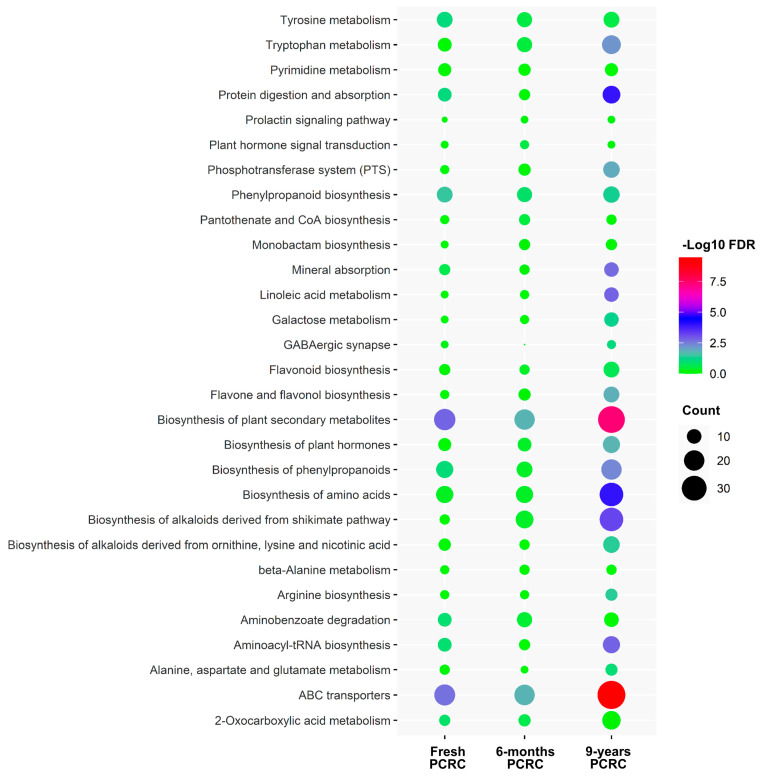
KEGG enrichment analysis of metabolites differently abundant between healthy and Huanglongbing (HLB)−affected pericarps of *Citrus reticulata* Blanco cv. Chachiensis (PCRC). The vertical coordinates represent the enriched pathways. The size of each point represents the number of differential metabolites in the pathway, and the color of the point represents the *p*-value.

KEGG pathway enrichment analysis of the significantly differential metabolites showed that the biosynthesis of plant secondary metabolites and ABC transporters was mainly altered in HLB-affected PCRC from three sources (Figure 4). The number of metabolites enriched in the biosynthesis of plant secondary and ABC transporters was higher in HLB-affected 9-year PCRC than those identified in HLB-affected fresh and 6-month PCRC (Figure 4 and Table S3). It was found that metabolites involved in the biosynthesis of amino acids, alkaloids, and phenylpropanoids were also enriched in HLB-affected 9-year PCRC (Figure 4). Flavonoid biosynthesis, 2-Oxocarboxylic acid metabolism, and linoleic acid metabolism were only significantly enriched in HLB-affected 9-year PCRC (Figure 4 and Table S3). In particular, most of the altered metabolites in flavonoid biosynthesis showed higher levels in HLB-affected 9-year PCRC than the healthy control (Table S3). 

To further analyze the effect of HLB on the metabolite profiles of PCRC during storage, the key compounds mainly involved in bioactivities and quality of PCRC were compared between healthy and HLB-affected PCRC at different aging times in the following sections. These compounds mainly included primary metabolites (amino acids, polysaccharides, fatty acids, and organic acids) and secondary metabolites (flavonoids, alkaloids, terpenoids, coumarins, and phenolic acids).

### 3.5. Alteration of Secondary Metabolites in PCRC by HLB

**Figure 5:**
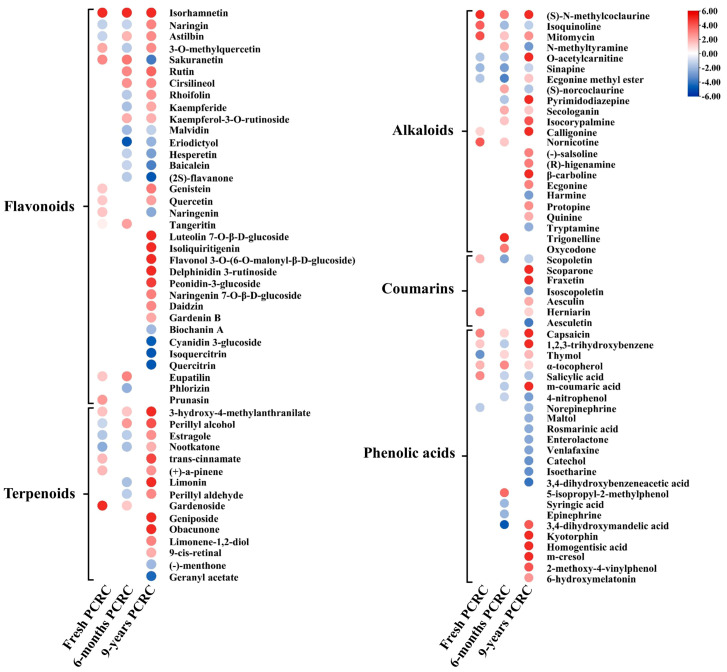
Heatmap of secondary metabolites differently abundant between healthy and Huanglongbing (HLB)−affected pericarp of *Citrus reticulata* Blanco cv. Chachiensis (PCRC) from three sources (fresh, 6 months, and 9 years).

A total of 103 altered secondary metabolites, including 34 flavonoids, 15 terpenoids, 23 alkaloids, 24 phenolic acids, and 7 coumarins, were identified by metabolite profiling (Figure 5). Overall, a higher number of secondary metabolites with increased levels were observed in HLB-affected 9-year PCRC as compared to HLB-affected 6-month and HLB-affected fresh PCRC (Figure 5). 

Among 34 altered flavonoids, 18 showed higher levels in HLB-affected 9-year PCRC than in healthy 9-year PCRC. Particularly, naringin, apiin, rhoifolin, kaempferide, luteolin 7-O-beta-D-glucoside, isoliquiritigenin, flavonol 3-O-(6-O-malonyl-beta-D-glucoside), delphinidin 3-rutinoside, peonidin-3-glucoside, naringenin 7-O-beta-D-glucoside, daidzin, and gardenin B were only accumulated in HLB-affected 9-year PCRC (Figure 5). Isorhamnetin was the only flavonoid increased in all three HLB-affected PCRC, which showed over 5-fold higher than the corresponding healthy control (Figure 5). Tangeritin only accumulated in HLB-affected fresh and 6-month PCRC, while astilbin, rutin, cirsilineol, and kaempferol-3-O-rutinoside were increased both in HLB-affected 6-month and 9-year PCRC (Figure 5). In addition, the content of sakuranetin, hesperetin, baicalein, (2S)-flavanone, naringenin, biochanin A, cyanidin 3-glucoside, isoquercitrin, and quercitrin decreased in HLB-affected PCRC as compared to healthy controls (Figure 5, Table S4).

Among 15 altered terpenoids, 13 were identified in HLB-affected 9-year PCRC, including 11 accumulated and two depleted (Figure 5). Particularly, nine out of 11 accumulated terpenoids were only identified in HLB-affected 9-year PCRC. Among these terpenoids, limonin and geniposide showed 22.6-fold and 16.2-fold higher levels in HLB-affected 9-year PCRC than in healthy 9-year PCRC, respectively. Among seven altered coumarins, three only accumulated in HLB-affected 9-year PCRC, including scoparone (61.8-fold), fraxetin (6.7-fold), and aesculin (2.1-fold) (Figure 5, Table S4). In contrast, the levels of scopoletin decreased in both HLB-affected 6-month and 9-year PCRC as compared to healthy controls (Figure 5).

Most altered alkaloid compounds significantly accumulated in all three HLB-affected PCRCs, including four increased in HLB-affected fresh PCRC, 5 increased in HLB-affected 6-month PCRC, and 12 increased in HLB-affected 9-year PCRC (Figure 5). Ten alkaloids were only significantly accumulated in HLB-affected 9-year PCRCs (Figure 5). Particularly, the pyrimidodiazepine, calligonine, and β-carboline showed 10.8-fold, 8.9-fold, and 8.9-fold higher HLB-affected 9-year PCRC than healthy controls, respectively (Figure 5, Table S4). The levels of (S)-N-methylcoclaurine and mitomycin were higher in all three types of HLB-affected PCRC than the corresponding healthy PCRC (Figure 5).

Of the 24 altered phenolic acids, 18 were identified in HLB-affected 9-year PCRC, while only 3 and 5 were identified in HLB-affected fresh PCRC and HLB-affected 6-month PCRC, respectively (Figure 5). The m-coumaric acid (15.2-fold), 3,4-dihydroxymandelic acid (4.0-fold), kyotorphin (23.8-fold), homogentisic acid (11.0-fold), m-cresol (6.8-fold), 2-methoxy-4-vinylphenol (4.1-fold), and 6-hydroxymelatonin (2.6-fold) were only accumulated in HLB-affected 9-year PCRC (Figure 5, Table S4). Two phenolic acids, capsaicin and α-tocopherol, showed higher levels in all three HLB-affected PCRCs compared to healthy controls (Figure 5). In addition to the phenolic acids with increased levels, nine phenolic acids showed lower levels in HLB-affected 9-year PCRC than in healthy controls, respectively (Figure 5). However, the decrease in these phenolic acids was not at a high level in HLB-affected 6-month PCRC and 9-year PCRC (Figure 5).

### 3.6. Changes in Primary Metabolites of PCRC Affected by HLB

**Figure 6:**
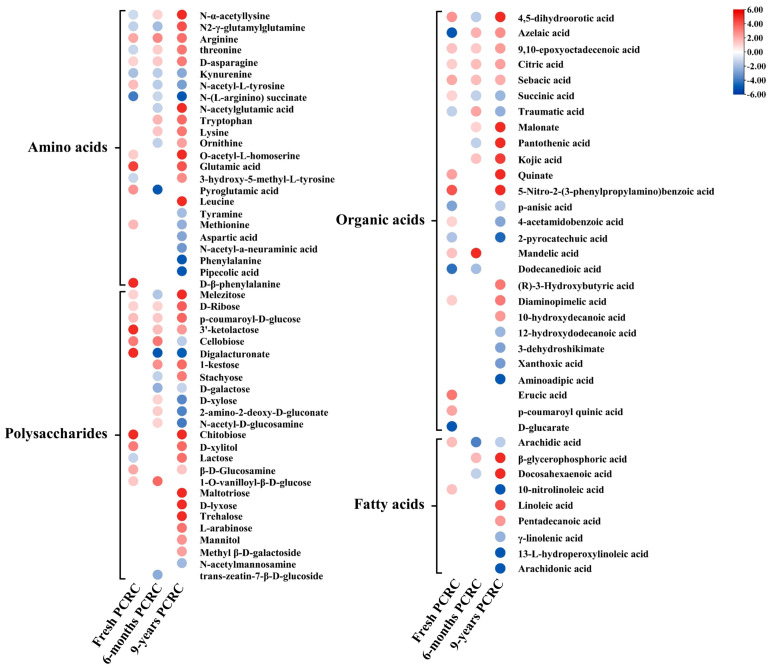
Heatmap of primary metabolites differently abundant between healthy and Huanglongbing (HLB)−affected pericarp of *Citrus reticulata* Blanco cv. Chachiensis (PCRC) from three sources (fresh, 6 months, and 9 years).

Comparative metabolome analysis identified a total of 87 altered primary metabolites between healthy PCRC and HLB-affected PCRC of three groups, including 25 amino acids, 25 polysaccharides, 28 organic acids, and 9 fatty acids (Figure 6). Compared to HLB-affected fresh PCRC and 6-month PCRC, most of the altered primary metabolites were identified in HLB-affected 9-year PCRC (Figure 6). 

Among 25 altered amino acids, 23 were identified in HLB-affected 9-year PCRC, including 14 increased and 9 decreased (Figure 6). The L-2-hydroxyglutaric acid, N-α-acetyllysine, N2-γ-glutamylglutamine, threonine, D-asparagine, N-acetylglutamic acid, tryptophan, lysine, ornithine, O-acetyl-L-homoserine, and leucine only showed significantly higher levels in HLB-affected 9-year PCRC as compared to HLB-affected fresh and 6-month PCRC (Figure 6). In addition, only five and two altered amino acids were identified in HLB-affected fresh PCRC and 6-month PCRC, respectively. It was found that the content of arginine was increased in all three types of HLB-affected PCRC as compared to the corresponding healthy control (Figure 6). In contrast, eight amino acids, including kynurenine, N-acetyl-L-tyrosine, N-(L-arginino) succinate, tyramine, methionine, aspartic acid, N-acetyl-a-neuraminic acid, phenylalanine, and pipecolic acid, were only depleted in HLB-affected 9-year PCRC as compared to healthy 9-year PCRC (Figure 6). 

Compared to healthy controls, a total of 15 polysaccharides showed increased levels in HLB-affected 9-year PCRC, while six and three polysaccharides showed higher levels in HLB-affected fresh and 6-month PCRC, respectively (Figure 6). Melezitose, D-ribose, p-coumaroyl-D-glucose, stachyose, lactose, maltotriose, D-lyxose, trehalose, L-arabinose, mannitol, and methyl β-D-galactoside were only accumulated in HLB-affected 9-year PCRC. It was also found that 3’-ketolactose, chitobiose, and D-xylitol showed increased levels in HLB-affected fresh and 9-year PCRC. Particularly, the chitobiose were 87.4-fold and 208.4-fold higher in HLB-affected fresh and 9-year PCRC than the corresponding healthy PCRC, respectively (Figure 6, Table S5). 

HLB-affected 9-year PCRC contained a higher number of altered organic acids than HLB-affected fresh and 6-month PCRC, including 13 showing higher levels and eight showing decreased levels as compared to healthy controls. In particular, malonate (150.3-fold), pantothenic acid (66.5-fold), 4-guanidinobutanoic acid (17.4-fold), 4,5-dihydroorotic acid (11.7-fold), and quinate (10.4-fold) showed relatively higher levels in HLB-affected 9-year PCRC than healthy 9-year PCRC (Figure 6, Table S5). In contrast, the levels of succinic acid, traumatic acid, 4-acetamidobenzoic acid, 2-pyrocatechuic acid, 12-hydroxydodecanoic acid, 3-dehydroshikimate, xanthoxic acid, and aminoadipic acid were only decreased in HLB-affected 9-year PCRC (Figure 6). Citric acid and sebacic acid showed higher levels in all three types of HLB-affected PCRC as compared to healthy controls (Figure 6). 

Among nine altered fatty acids, eight were identified in HLB-affected 9-year PCRC, while only one altered fatty acid was identified in HLB-affected 6-month PCRC, and none were found in HLB-affected fresh PCRC (Figure 6). Of eight altered fatty acids, four (β-glycerophosphoric acid, docosahexaenoic acid, linoleic acid, and pentadecanoic acid) showed increased levels, and four (10-nitrolinoleic acid, γ-linolenic acid, 13-L-hydroperoxylinoleic acid, and arachidonic acid) showed decreased levels (Figure 6). The level of beta-glycerophosphoric acid was 13.0-fold higher in HLB-affected 9-year PCRC than in healthy controls (Figure 6, Table S5). However, arachidonic acid, 10-nitrolinoleic acid, and 13-L-hydroperoxylinoleic acid showed 50.0-fold, 33.3-fold, and 10.0-fold lower levels in HLB-affected 9-year PCRC as compared to healthy controls, respectively (Figure 6).

### 3.7. TFs Content, TPs Content and TAC of HLB-Affected and Healthy PCRC

**Figure 7:**
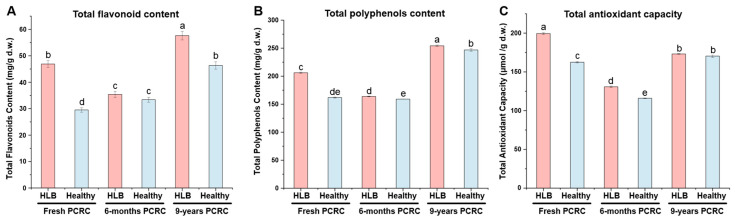
Determination results of total flavonoid content (**A**), total polyphenol content (**B**), and total antioxidant capacity (**C**) between healthy and Huanglongbing (HLB)-affected pericarps of Citrus reticulata Blanco cv. Chachiensis (PCRC). Fresh: fresh PCRC. 6 months: sun-dried PCRC that was stored for six months. 9 years: sun-dried PCRC that was stored for nine years. Different letters denoted significant differences (*p* < 0.05) among the six groups.

As shown in Figure 7, the TFs content of HLB-affected fresh PCRC and HLB-affected 9-year PCRC was significantly higher than those identified in the corresponding healthy PCRC (*p* < 0.05), while no difference was observed between HLB-affected 6-month and healthy 6-month PCRC (Figure 7A). Among three types of HLB-affected PCRC, HLB-affected 9-year-olds had the highest TF contents with an average value of 57.6 ± 1.6 mg/g d.w., followed by HLB-affected fresh PCRC (46.9 ± 1.3 mg/g d.w.) and HLB-affected 6-month PCRC (35.4 ± 1.1 mg/g d.w.) (Figure 7A). This indicated that the HLB caused the accumulation of TP content in fresh PCRC and maintained a higher level in HLB-affected PCRC than healthy PCRC after long-term storage. The same trend was observed in the TP contents of HLB-affected and healthy PCRC from three sources (Figure 7B). HLB-affected 9-year PCRC contained significantly higher TP contents (254.3 ± 1.4 mg/g d.w.) than HLB-affected fresh PCRC (206.1 ± 1.1 mg/g d.w.) and HLB-affected 6-month PCRC (163.8 ± 0.6 mg/g d.w.) (*p* < 0.05) (Figure 7B). The TP contents of HLB-affected fresh and 9-year PCRC were also significantly higher than those identified in the corresponding healthy control (*p* < 0.05) (Figure 7B). 

Evaluation of TAC with the FRAP method showed that the FRAP value of HLB-affected fresh PCRC (199.4 ± 1.2 μmol/g d.w.) was highest among three HLB-affected PCRC, followed by HLB-affected 9-year PCRC (173.2 ± 0.5 μmol/g d.w.) and HLB-affected 6-month PCRC (130.8 ± 1.0 μmol/g d.w.) (Figure 7C). Both HLB-affected fresh PCRC and HLB-affected 6-month PCRC showed significantly higher FRAP values than healthy controls (*p* < 0.05) (Figure 7C). However, the FRAP value of HLB-affected 9-year PCRC was slightly higher than that of healthy 9-year PCRC (170.4 ± 1.8 μmol/g d.w.) but with no significant difference (Figure 7C). The varied antioxidant capacity observed between HLB-affected PCRC and healthy PCRC from three sources indicated that HLB did not affect the antioxidant capacity of long-term stored PCRC but increased the antioxidant capacity of fresh PCRC and short-term stored storage.

## 4. Discussion

Plants produce various types of primary and secondary metabolites to protect themselves from biotic stresses. Research on the metabolic response of citrus plants against HLB has revealed that the secondary metabolites known for their antibacterial activity were not only induced by HLB/CLas infection but also correlated with citrus tolerance against CLas [20]. In this study, secondary metabolites were mainly accumulated in HLB-affected PCRC as compared to healthy PCRC, particularly in HLB-affected 9-year PCRC. This finding was consistent with the results of previous studies showing that CLas infection led to an increase in secondary metabolites in citrus plants. CLas infection caused the elevation of total flavonoids and total polyphenol levels in HLB-affected PCRC, particularly in HLB-affected 9-year PCRC. A previous study showed that CLas infection caused the accumulation of most flavonoids in Hamlin oranges and Murcott mandarins [20]. The phenolic compounds, mainly flavonoids and phenolic acids, were thought to be highly correlated with the bioactivity and quality of PCRC during storage [4]. The significantly higher levels of TFs (total flavonoids) and TPs (total polyphenols) contents in HLB-affected 9-year PCRC suggested that the bioactivity of HLB-affected 9-year PCRC may increase. In addition, both HLB-affected fresh and 6-month PCRC exhibited higher TAC levels than healthy controls, but there was no difference in TAC (total antioxidant capacity) levels between HLB-affected 9-year PCRC and healthy controls (Figure 7). The varied antioxidant capacity observed between HLB-affected PCRC and healthy PCRC from three sources indicated that HLB did not affect the antioxidant capacity of long-term stored PCRC but increased the antioxidant capacity of fresh and short-term stored PCRC.

Flavonoids, regarded as one of the main biological compounds in PCRC, possess various bioactivities, including antioxidant, anti-carcinogenic, and anti-inflammatory activities [5,6]. HLB caused the accumulation of flavonoids in HLB-affected PCRC, particularly in the 9-year PCRC (Figure 5). Among the accumulated flavonoids, naringin, the key active flavonoid component of PCRC, was reported to have broad-spectrum pharmacological and therapeutic properties in the treatment of various diseases, such as chronic liver injury, lung injury, and bowel disease [21]. Isorhamnetin has documented extensive pharmacological activities, including cardiovascular and cerebrovascular protection, anti-inflammatory, antitumor, and prevention of obesity [22]. Luteolin 7-O-beta-D-glucoside, which exhibited 19.3-fold higher levels in HLB-affected 9-year PCRC than in healthy controls, was found to have anti-inflammatory and active biological properties [23]. Isoliquiritigenin, a highly accumulated flavonoid acid (12.1-fold higher) in HLB-affected 9-year PCRC (Figure 5), demonstrated high antitumor efficacy [24]. The flavonoids had been shown to accumulate significantly in HLB-affected Hamlin oranges and Murcott mandarins as a response to CLas infection and played an important role in plant defense against CLas [20]. The increased contents of most bioactive flavonoids in HLB-affected 9-year PCRC indicated they should be induced by HLB and accumulated during the storage process, which contributes to the health benefits of long-term stored HLB-affected PCRC.

Terpenoids were the main volatile compounds identified in the essential oil of PCRC and possessed various biological activities, such as antioxidant, antimicrobial, anticancer, and antiallergic [25,26]. Most of the accumulated HLB-affected PCRC had been found to display numerous pharmacological activities. Citrus limonin, highly accumulated in HLB-affected 9-year PCRC, possessed anticancer, anti-inflammatory, analgesic, antibacterial, and anti-insect activity [27]. Geniposide, a well-known iridoid glycoside compound, exhibited a wide spectrum of pharmacological effects, such as antidiabetic, neuroprotective, anti-inflammatory, analgesic, antidepressant-like, antioxidant, antitumoral, and antithrombotic effects [28]. The increased number of bioactive terpenoids in HLB-affected 9-year PCRC indicated they could contribute to the biological and pharmacological activity and enhance the beneficial effect on health care of HLB-affected long-term stored PCRC.

Alkaloids and coumarins were the other two main bioactive ingredients identified in PCRC during storage, which both exhibited strong antioxidant and pharmacological activities [26,29]. A higher number of alkaloids and coumarins were accumulated in HLB-affected 9-year PCRC as compared to HLB-affected fresh and 6-month PCRC. Among the accumulated alkaloids, pyrimidodiazepine and β-carboline both displayed significant wide-spectrum activities, including anticancer, anti-inflammatory, antimicrobial, and antiviral activities [30,31]. Mitomycin, naturally produced by Streptomyces species, is an antibiotic that has been shown to have antitumor activity and is widely used in cancer chemotherapy [32]. A previous study had shown that HLB induced the enrichment of *Streptomyces* in infected plant tissue [33]. The accumulation of mitomycin in HLB-affected PCRC could be highly correlated with the enrichment of *Streptomyces* in PCRC caused by CLas infection. In addition, four out of nine altered coumarins (scoparone, fraxetin, aesculin, and herniarin) were accumulated in HLB-affected 9-year PCRCs. Both scoparone (61.8-fold) and fraxetin possessed versatile activities, such as antioxidant, antifibrotic, anti-apoptotic, antifibrotic, and anti-inflammatory activities [34,35]. Therefore, the high increase in the level of most alkaloids and coumarins with various bioactivities in HLB-affected dried PCRC suggested that they may contribute to the pharmacological efficacy of HLB-affected 9-year PCRC, particularly for the long-term stored HLB-affected PCRC.

Phenolic acid had been reported as the main component increased in PCRC during the aging process, which was regarded as a possible indicator of PCRC quality and showed antioxidant, antibacterial, anticancer, and anti-inflammatory activities [7,36]. m-Coumaric acid, which possessed high antioxidant activity [37], was highly increased in HLB-affected 9-year PCRC. Capsaicin, accumulated in all three types of HLB-affected PCRC, was known to exhibit antioxidative, hypotensive, antimicrobial, and anticancer effects [38]. Compared to HLB-affected fresh PCRC and HLB-affected 6-month PCRC, the increased number of accumulated bioactive phenolic acids identified in HLB-affected 9-year PCRC suggested that the HLB could enhance the bioactivity of long-term stored HLB-affected PCRC.

Amino acids were the main types of active compounds found in dried PCRC with different aging years and had an influence on the quality of PCRC [7,33]. A previous study found that most amino acids in PCRC showed decreased trends during aging processes, while some amino acids, such as valine, isoleucine, aspartate, threonine, leucine, and glutamine, increased at a certain or most aging time [7]. In this study, the accumulation of threonine and leucine in HLB-affected 9-year PCRC may contribute to the quality of PCRC (Figure 6). In addition, asparagine, which increased in HLB-affected 9-year PCRC (Figure 6), was identified as one of the most critical marker compounds responsible for distinguishing the storage years of PCRC and showed potential antioxidation activity [39]. Studies have shown that HLB greatly affects the biosynthesis of amino acids in citrus [20,40]. In particular, stress-related amino acids such as proline, serine, threonine, phenylalanine, valine, and isoleucine are significantly elevated in HLB-affected citrus plants [20,40]. Based on the dynamic change in amino acids observed in three types of HLB-affected PCRC, the increased number of accumulated amino acids in the HLB-affected 9-year PCRC may improve the health benefits of HLB-affected 9-year PCRC.

Polysaccharides were important bioactive chemicals in PCRC and had obvious antioxidant properties and immunomodulatory activity [41]. HLB caused the accumulation of most polysaccharides in HLB-affected PCRC, particularly the HLB-affected 9-year PCRC (Figure 6). Among these accumulated polysaccharides, chitobiose, an effective antioxidant with the ability to scavenge superoxide radicals [42], was 87.4-fold and 208.4-fold higher in HLB-affected fresh and 9-year PCRC than healthy controls, respectively (Figure 6). The high accumulation of chitobiose in HLB-affected 9-year PCRC suggested it could significantly increase the antioxidant capacity of dried HLB-affected PCRC. In addition, a recent study found that HLB caused a significant accumulation of maltotriose and melezitose in citrus plants [43]. Both maltotriose (8.1-fold) and melezitose (10.8-fold) showed higher levels in HLB-affected 9-year PCRC than in healthy controls (Figure 6). The increase in polysaccharides in HLB-affected PCRC suggested that they could enhance the bioactivities of HLB-affected PCRC, particularly the long-term storage of HLB-affected PCRC.

Both organic acids and fatty acids were the main bioactive metabolites accumulated in PCRC during aging processing [26,39]. In this study, a higher number of accumulated organic acids and fatty acids were identified in HLB-affected 9-year PCRC than in HLB-affected fresh PCRC and 6-month PCRC, indicating an improved antioxidant capacity by the accumulation of bioactive organic acids and fatty acids in HLB-affected long-term stored PCRC. Among the accumulated organic acids, malonate and pantothenic acid were highly accumulated in HLB-affected 9-year PCRC as compared to healthy PCRC (Figure 6, Table S5). A previous study had shown that malonate was a competitive inhibitor of succinate dehydrogenase and showed promise in ameliorating ischemia/reperfusion injury under a lowered pH [44]. Pantothenic acid (also called vitamin B5) is an essential vitamin that has some health benefits for humans [45]. The increase in organic acids with bioactive properties could improve the healthcare effects of HLB-affected 9-year PCRC. In contrast, several organic acids and fatty acids showed decreased levels in HLB-affected 9-year PCRC as compared to healthy PCRC. None of the depleted organic acids in the 9-year PCRC were identified as the key compounds, according to previous studies [26,39]. This indicated that most key organic acids that contributed to the bioactivity of PCRC were not influenced by HLB. Three fatty acids, i.e., arachidonic acid, 10-nitrolinoleic acid, and 13-L-hydroperoxylinoleic acid, strongly decreased in HLB-affected 9-year PCRC as compared to healthy PCRC. Arachidonic acid was known as a critical precursor in the biosynthesis of prostanoids, thromboxanes, and leukotrienes, which played important roles in the initiation and development of human diseases, including cardiovascular, cancer, and inflammatory diseases [24]. The significant decrease in the level of arachidonic acid in HLB-affected 9-year PCRC could reduce the risk of arachidonic acid-derived disease, which in turn improves the health benefits of PCRC.

## 5. Conclusions

Our study investigated the metabolic changes of PCRC affected by HLB by screening and analyzing healthy and HLB-affected PCRC from three sources (fresh, 6 months, and 9 years) using untargeted LC–MS. HLB has a significant impact on the quality of PCRC, particularly in long-term stored samples. The increased levels of bioactive metabolites in HLB-affected 9-year-old PCRC, including flavonoids, terpenoids, alkaloids, amino acids, polysaccharides, and organic acids, suggested potential biological activity. Further research could focus on investigating the specific pathways and enzymes involved in the biosynthesis and accumulation of these metabolites in HLB-affected PCRC. This could help identify potential targets for developing strategies to enhance the bioactive properties of PCRC. Thus, this study provided details on the metabolic changes of PCRC affected by HLB and put forward the potential biological activity of long-term stored PCRC induced by HLB, although the safety and efficacy of using HLB-affected PCRC in these applications required further assessment.

## Data Availability

The original contributions presented in the study are included in the article/Supplementary Material. Metabolome analysis data have been deposited in the Metabolights public repository under accession number MTBLS9210. Further inquiries can be directed to the corresponding authors.

## Supplementary Materials

The following supporting information can be downloaded at: https://www.mdpi.com/article/10.3390/foods13060827/s1, Figure S1: Heat map of healthy and HLB-affected PCRC samples from three sources (fresh, 6 months, and 9 years); Table S1: Primer sets used in this study; Table S2: Real-time PCR result of all PCRC samples with primer set targeted “Candidatus Liberibacter asiaticus” genes (16S rRNA genes: CLas4G/HLBr and RNR genes: RNRf/RNRr) and citrus plant genes (18S rRNA genes:18S-F/18S-R); Table S3: The significant KEGG pathways based on metabolites with significantly differential abundance in HLB-affected and healthy PCRC of three sources (fresh, 6 months, and 9 years); Table S4: Changes in secondary metabolites in HLB-affected and healthy PCRC of three sources (fresh, 6 months, and 9 years); Table S5: Changes in primary metabolites in HLB-affected and healthy PCRC of three sources (fresh, 6 months, and 9 years).
