# Supplementary material for: Metabolomic Profiling Reveals the Quality Variations in Citri Reticulatae Pericarpium (Citrus reticulata Blanco cv. Chachiensis) with Different Storage Ages in Response to “Candidatus Liberibacter Asiaticus” Infection
Source: Foods. 2024 Mar 8;13(6):827. doi: 10.3390/foods13060827 (PMC10969107; doi:10.3390/foods13060827)
Supplement: Supplementary file 1 [file foods-13-00827-s001.zip › foods-2884644-supplementary.pdf]

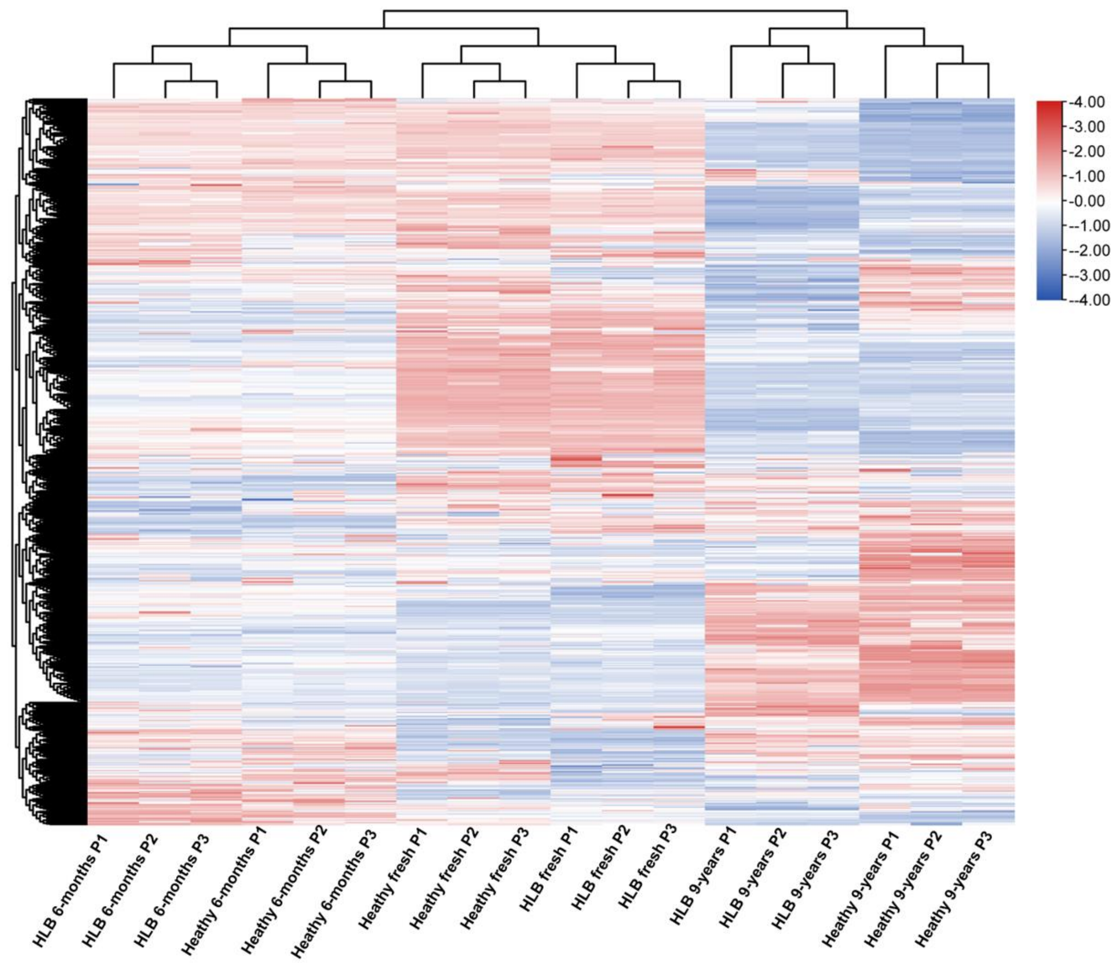

Figure S1. Heat map of healthy and HLB-affected PCRC samples from three sources (fresh, 6-months and 9-years).

Table S1. Primer sets used in this study

Name	Type	Sequence (5’-3’)	Amplicon size (bp)	Target gene	Reference
18S-F	Forward	GCAACGGATATCTCGGCTCT	100	18S rRNA genes of citrus	Yan et al., 2012
18S-R	Reverse	TGCGTTCAAAGACTCGATGG			
RNRf	Forward	CATGCTCCATGAAGCTACCC	75	nrdB genes of CLas	Zheng et al., 2016
RNRr	Reverse	GGAGCATTTAACCCCACGAA			
CLas4G	Forward	AGTCGAGCGCGTATGCGAAT	80	16S rRNA genes of CLas	Bao et al., 2020
HLBr	Reverse	GCGTTATCCCGTAGAAAAAGGTAG			

Table S2. Real-time PCR result of all PCRC samples with primer set targeted "Candidatus Liberibacter asiaticus" genes (16S rRNA genes: CLas4G/HLBr and RNR genes: RNRf/RNRr) and citrus plant genes (18S rRNA genes:18S-F/18S-R)

Sample type	subgroup	Sample ID	Ct (CLas4G/HLBr)	Ct (RNRf/RNRr)	Ct (18S-F/18S-R)
9-years PCRC	HLB-candidate	2012-D1	28.10	27.62	22.18
	HLB-candidate	2012-D2	28.27	27.38	22.28
	HLB-candidate	2012-D3*	25.87	25.35	21.98
	HLB-candidate	2012-D4*	25.33	24.21	20.16
	HLB-candidate	2012-D5*	27.68	26.84	21.73
	Healthy	2012-H1	N/A	N/A	20.09
	Healthy	2012-H2	N/A	N/A	27.70
	Healthy	2012-H3	N/A	N/A	27.33
	Healthy	2012-H4	N/A	N/A	26.77
	Healthy	2012-H5	N/A	N/A	27.81
	Healthy	2012-H6	N/A	N/A	23.53
	Healthy	2012-H7	N/A	N/A	26.85
	Healthy	2012-H8	N/A	N/A	27.61
	Healthy	2012-H9	N/A	N/A	24.05
	Healthy	2012-H10	N/A	N/A	24.30
	Healthy	2012-H11	N/A	N/A	27.49
	Healthy	2012-H12	N/A	N/A	25.78
	Healthy	2012-H13	N/A	N/A	27.04
	Healthy	2012-H14	N/A	N/A	29.66
	Healthy	2012-H15	N/A	N/A	26.40
	Healthy	2012-H16	N/A	N/A	20.66
	Healthy	2012-H17	35.15	N/A	23.57
	Healthy	2012-H18	N/A	N/A	21.66
	Healthy	2012-H19	37.65	N/A	20.82
	Healthy	2012-H20	N/A	N/A	19.04
	Healthy	2012-H21	N/A	N/A	22.59
	Healthy	2012-H22	N/A	N/A	21.22
	Healthy	2012-2-12	N/A	N/A	20.74
6-months PCRC	HLB-candidate	2021-D1	22.50	20.93	13.59
	HLB-candidate	2021-D2*	16.29	15.48	13.61
	HLB-candidate	2021-D3*	17.21	16.27	15.60
	HLB-candidate	2021-D4	20.67	20.22	14.56
	HLB-candidate	2021-D5	24.18	23.42	15.19
	HLB-candidate	2021-D6	24.19	23.34	17.43
	HLB-candidate	2021-D7*	18.34	17.76	14.67
	HLB-candidate	2021-D8	18.22	17.41	13.15
	Healthy	2021-H1*	N/A	N/A	17.07
	Healthy	2021-H2	N/A	N/A	18.84
	Healthy	2021-H3*	N/A	N/A	15.01

	Healthy	2021-H4	N/A	N/A	19.82
	Healthy	2021-H5*	N/A	N/A	15.95
	Healthy	2021-H6	N/A	N/A	20.97
	Healthy	2021-H7	N/A	N/A	36.69
	Healthy	2021-H8	N/A	N/A	14.54
Fresh PCRC	HLB-candidate	2021-FD1*	15.58	15.33	14.33
	HLB-candidate	2021-FD2	15.69	14.97	14.09
	HLB-candidate	2021-FD3*	15.70	15.40	13.70
	HLB-candidate	2021-FD4	15.49	14.96	13.60
	HLB-candidate	2021-FD5	16.37	15.71	13.77
	HLB-candidate	2021-FD6*	15.73	14.96	13.51
	HLB-candidate	2021-FD7	15.78	15.87	13.92
	HLB-candidate	2021-FD8	17.76	16.83	13.59
	Healthy	2021-FH1	N/A	N/A	18.41
	Healthy	2021-FH2*	N/A	N/A	17.45
	Healthy	2021-FH3	N/A	N/A	19.50
	Healthy	2021-FH4	N/A	N/A	19.69
	Healthy	2021-FH5*	N/A	N/A	18.78
	Healthy	2021-FH6	N/A	N/A	18.36
	Healthy	2021-FH7*	N/A	N/A	14.15
	Healthy	2021-FH8	38.07	N/A	22.11

*Selected PCRC samples for LC-MS analyses

Table S3. The significant KEGG pathways based on metabolites with significantly differential abundance in HLB-affected and healthy PCRC of three sources (fresh, 6-months and 9-years).

pathway_id	pathway_name	Hits	Up_hits	Down_hits	Pvalue	FDR
**HLB-affected fresh PCRC**						
map01060	Biosynthesis of plant secondary metabolites	22	15	7	5.64E-06	1.53E-03
map02010	ABC transporters	21	11	10	1.23E-05	2.21E-03
map01061	Biosynthesis of phenylpropanoids	14	10	4	1.21E-03	9.34E-02
map01230	Biosynthesis of amino acids	14	7	7	9.00E-03	4.87E-01
map00940	Phenylpropanoid biosynthesis	12	8	4	2.43E-04	3.29E-02
map00350	Tyrosine metabolism	12	9	3	8.91E-04	8.03E-02
map04974	Protein digestion and absorption	9	4	5	7.86E-04	8.03E-02
map00970	Aminoacyl-tRNA biosynthesis	9	4	5	1.67E-03	1.13E-01
map00380	Tryptophan metabolism	9	6	3	3.45E-02	9.83E-01
map00240	Pyrimidine metabolism	8	4	4	2.31E-02	9.28E-01
map01070	Biosynthesis of plant hormones	8	8	0	2.95E-02	9.55E-01
map00360	Phenylalanine metabolism	7	7	0	4.20E-02	1.00E+00
map04978	Mineral absorption	6	2	4	3.96E-03	2.38E-01
map00250	Alanine, aspartate and glutamate metabolism	5	3	2	1.59E-02	7.15E-01
map01065	Biosynthesis of alkaloids derived from histidine and purine	5	4	1	3.85E-02	1.00E+00
map00220	Arginine biosynthesis	4	2	2	3.30E-02	9.83E-01
map04727	GABAergic synapse	3	2	1	1.03E-02	5.09E-01
map04075	Plant hormone signal transduction	3	2	1	2.40E-02	9.28E-01
map00471	D-Glutamine and D-glutamate metabolism	3	1	2	3.00E-02	9.55E-01
map07226	Progesterone, androgen and estrogen receptor agonists/antagonists	2	1	1	2.62E-02	9.45E-01
**HLB-affected 6-months PCRC**						
map02010	ABC transporters	20	11	9	3.93E-05	1.62E-02
map01060	Biosynthesis of plant secondary metabolites	20	16	4	5.98E-05	1.62E-02
map01063	Biosynthesis of alkaloids derived from shikimate pathway	15	12	3	9.89E-03	4.28E-01
map01230	Biosynthesis of amino acids	14	11	3	8.78E-03	4.28E-01
map01061	Biosynthesis of phenylpropanoids	12	5	7	9.12E-03	4.28E-01
map00940	Phenylpropanoid biosynthesis	11	4	7	9.22E-04	1.25E-01
map00350	Tyrosine metabolism	11	4	7	2.89E-03	2.63E-01
map00380	Tryptophan metabolism	11	6	5	4.72E-03	3.19E-01
map00627	Aminobenzoate degradation	11	6	5	6.19E-03	3.72E-01
map01070	Biosynthesis of plant hormones	9	5	4	1.03E-02	4.28E-01
map05230	Central carbon metabolism in cancer	8	6	2	6.45E-04	1.16E-01
map00944	Flavone and flavonol biosynthesis	7	2	5	1.87E-02	5.96E-01
map02060	Phosphotransferase system (PTS)	7	5	2	3.25E-02	8.85E-01
map00360	Phenylalanine metabolism	7	3	4	4.15E-02	9.35E-01
map00770	Pantothenate and CoA biosynthesis	6	1	5	4.66E-03	3.19E-01

map00261	Monobactam biosynthesis	6	6	0	1.71E-02	5.96E-01
map04974	Protein digestion and absorption	6	6	0	3.92E-02	9.23E-01
map04978	Mineral absorption	5	4	1	1.81E-02	5.96E-01
map00410	beta-Alanine metabolism	5	1	4	2.69E-02	8.09E-01
map00030	Pentose phosphate pathway	5	4	1	3.81E-02	9.23E-01
map04075	Plant hormone signal transduction	4	2	2	2.92E-03	2.63E-01
map00220	Arginine biosynthesis	4	1	3	3.27E-02	8.85E-01
map04917	Prolactin signaling pathway	3	1	2	1.86E-02	5.96E-01
map02030	Bacterial chemotaxis	2	1	1	3.77E-02	9.23E-01
**HLB-affected 9-years PCRC**						
map02010	ABC transporters	38	24	14	6.20E-13	3.35E-10
map01060	Biosynthesis of plant secondary metabolites	35	19	16	1.62E-10	4.38E-08
map01230	Biosynthesis of amino acids	27	14	13	7.70E-07	1.04E-04
map01063	Biosynthesis of alkaloids derived from shikimate pathway	27	14	13	7.25E-06	6.54E-04
map01061	Biosynthesis of phenylpropanoids	20	8	12	7.43E-05	4.02E-03
map00380	Tryptophan metabolism	17	9	8	1.29E-04	6.35E-03
map01210	2-Oxocarboxylic acid metabolism	16	9	7	4.63E-02	6.11E-01
map04974	Protein digestion and absorption	15	9	6	1.00E-06	1.09E-04
map00970	Aminoacyl-tRNA biosynthesis	14	9	5	2.11E-05	1.48E-03
map01070	Biosynthesis of plant hormones	14	6	8	4.75E-04	1.71E-02
map02060	Phosphotransferase system (PTS)	13	5	8	2.62E-04	1.18E-02
map01064	Biosynthesis of alkaloids derived from ornithine, lysine and nicotinic acid	13	8	5	1.35E-03	4.28E-02
map00940	Phenylpropanoid biosynthesis	13	7	6	1.55E-03	4.66E-02
map00944	Flavone and flavonol biosynthesis	12	9	3	3.30E-04	1.37E-02
map00941	Flavonoid biosynthesis	12	7	5	9.24E-03	2.00E-01
map00350	Tyrosine metabolism	12	3	9	1.39E-02	2.52E-01
map00591	Linoleic acid metabolism	10	5	5	2.19E-05	1.48E-03
map04978	Mineral absorption	10	6	4	3.11E-05	1.87E-03
map00052	Galactose metabolism	10	7	3	1.96E-03	5.58E-02
map00360	Phenylalanine metabolism	10	6	4	1.40E-02	2.52E-01
map04080	Neuroactive ligand-receptor interaction	9	4	5	1.52E-02	2.65E-01
map00400	Phenylalanine, tyrosine and tryptophan biosynthesis	8	2	6	3.25E-03	8.37E-02
map00220	Arginine biosynthesis	7	4	3	1.17E-03	3.96E-02
map00250	Alanine, aspartate and glutamate metabolism	7	3	4	4.06E-03	9.97E-02
map04742	Taste transduction	7	5	2	8.85E-03	2.00E-01
map00300	Lysine biosynthesis	6	2	4	4.59E-02	6.11E-01
map01065	Biosynthesis of alkaloids derived from histidine and purine	6	3	3	4.59E-02	6.11E-01
map00290	Valine, leucine and isoleucine biosynthesis	5	4	1	2.65E-02	4.35E-01

map01053	Biosynthesis of siderophore group nonribosomal peptides	5	2	3	3.15E-02	4.87E-01
map04024	cAMP signaling pathway	5	3	2	3.70E-02	5.41E-01
map04973	Carbohydrate digestion and absorption	5	2	3	4.97E-02	6.25E-01
map02030	Bacterial chemotaxis	4	1	3	4.36E-04	1.68E-02
map04727	GABAergic synapse	4	2	2	3.04E-03	8.23E-02
map04721	Synaptic vesicle cycle	4	3	1	9.95E-03	2.07E-01
map05030	Cocaine addiction	3	1	2	1.21E-02	2.34E-01
map05033	Nicotine addiction	3	3	0	1.21E-02	2.34E-01
map05031	Amphetamine addiction	3	1	2	2.59E-02	4.35E-01
map05034	Alcoholism	3	1	2	3.49E-02	5.25E-01
map04917	Prolactin signaling pathway	3	1	2	4.54E-02	6.11E-01
map07110	Benzoic acid family	2	1	1	5.76E-03	1.35E-01
map04150	mTOR signaling pathway	2	2	0	3.12E-02	4.87E-01
map07226	Progesterone, androgen and estrogen receptor agonists/antagonists	2	2	0	4.94E-02	6.25E-01

Table S4. Changes in secondary metabolites in HLB-affected and healthy PCRC of three sources (fresh, 6-months and 9-years).

Name / Type	Fold Change (p-value < 0.05 and VIP > 1)		
	fresh PCRC	6-months PCRC	9-years PCRC
**Flavonoids**			
Isorhamnetin	7.0	5.0	10.4
Naringin	-1.4	-1.3	3.0
Astilbin	-1.3	1.8	2.8
3-O-methylquercetin	2.1	-1.6	2.8
Sakuranetin	2.9	3.3	-4.0
Rutin	NA	2.9	3.7
Cirsilineol	NA	2.7	2.9
Rhoifolin	NA	-1.6	2.6
Kaempferide	NA	-1.9	2.1
Kaempferol-3-O-rutinoside	NA	2.0	1.9
Malvidin	NA	-2.1	-1.4
Eriodictyol	NA	-5.9	-2.3
Hesperetin	NA	-1.4	-2.9
Baicalein	NA	-1.3	-3.8
(2S)-flavanone	NA	-1.6	-5.3
Genistein	1.3	NA	3.2
Quercetin	1.3	NA	2.3
Naringenin	1.4	NA	-2.6
Tangeritin	0.4	2.3	NA
Luteolin 7-O-β-D-glucoside	NA	NA	19.3
Isoliquiritigenin	NA	NA	12.1
Flavonol 3-O-(6-O-malonyl-β-D-glucoside)	NA	NA	9.2
Delphinidin 3-rutinoside	NA	NA	5.9
Peonidin-3-glucoside	NA	NA	4.6
Naringenin 7-O-β-D-glucoside	NA	NA	3.1
Daidzin	NA	NA	2.9
Gardenin B	NA	NA	2.1
Biochanin A	NA	NA	-2.1
Cyanidin 3-glucoside	NA	NA	-4.8
Isoquercitrin	NA	NA	-5.3
Quercitrin	NA	NA	-5.3
Eupatilin	1.4	3.0	NA
Phlorizin	NA	-2.4	NA
Prunasin	2.5	NA	NA
**terpenoids**			
3-hydroxy-4-methylanthranilate	1.5	1.4	7.9
Perillyl alcohol	-1.2	2.5	4.2
Estragole	-1.7	-1.5	2.7

Nootkatone	-2.5	-1.9	1.9
trans-cinnamate	1.7	NA	4.4
(+)-a-pinene	1.7	NA	2.6
Limonin	NA	-1.9	22.6
Perillyl aldehyde	NA	-1.5	2.9
Gardenoside	10.4	1.3	NA
Geniposide	NA	NA	16.2
Obacunone	NA	NA	5.2
Limonene-1,2-diol	NA	NA	3.0
9-cis-retinal	NA	NA	2.0
(-)-menthone	NA	NA	-2.1
Geranyl acetate	NA	NA	-4.5
**Alkaloids**			
(S)-N-methylcoclaurine	5.9	3.1	5.0
Isoquinoline	3.9	-1.9	-1.3
Mitomycin	4.2	1.5	2.7
N-methyltyramine	NA	2.0	-2.9
O-acetylcarnitine	-1.6	-1.7	7.3
Sinapine	-2.0	-2.8	-1.2
Ecgonine methyl ester	-1.6	-3.6	1.5
(S)-norcoclaurine	NA	2.2	-1.6
Pyrimidodiazepine	NA	-1.6	10.8
Secologanin	NA	2.1	1.4
Isocorypalmine	NA	1.5	4.1
Calligonine	1.2	NA	8.9
Nornicotine	4.0	1.4	NA
(-)-salsoline	NA	NA	3.1
(R)-higenamine	NA	NA	3.2
β-carboline	NA	NA	8.9
Ecgonine	NA	NA	3.1
Harmine	NA	NA	-2.8
Protopine	NA	NA	2.8
Quinine	NA	NA	2.1
Tryptamine	NA	NA	-2.3
Trigonelline	NA	5.0	NA
Oxycodone	NA	3.3	NA
**Coumarins**			
Scopoletin	1.9	-2.6	-1.4
Scoparone	NA	NA	61.8
Fraxetin	NA	NA	6.7
Isoscopoletin	NA	NA	-2.8
Aesculin	NA	NA	2.1
Herniarin	2.9	NA	1.2
Aesculetin	NA	NA	-3.8

**Phenolic acids**			
Capsaicin	3.1	1.1	7.3
1,2,3-trihydroxybenzene	1.4	-1.4	6.5
Thymol	-3.1	1.1	1.8
α-tocopherol	1.9	2.9	1.2
Salicylic acid	2.9	-1.2	-1.7
m-coumaric acid	NA	-1.4	15.2
4-nitrophenol	NA	-1.2	-2.8
Norepinephrine	-1.4	NA	-2.0
Maltol	NA	NA	-2.2
Rosmarinic acid	NA	NA	-2.4
Enterolactone	NA	NA	-2.5
Venlafaxine	NA	NA	-2.6
Catechol	NA	NA	-3.1
Isoetharine	NA	NA	-3.2
3,4-dihydroxybenzeneacetic acid	NA	NA	-4.0
5-isopropyl-2-methylphenol	NA	3.6	NA
Syringic acid	NA	-2.0	NA
Epinephrine	NA	-2.1	NA
3,4-dihydroxymandelic acid	NA	-4.8	4.0
Kyotorphin	NA	NA	23.8
Homogentisic acid	NA	NA	11.0
m-cresol	NA	NA	6.8
2-methoxy-4-vinylphenol	NA	NA	4.1
6-hydroxymelatonin	NA	NA	2.6

[a]NA, No applicable

Table S5. Changes in primary metabolites in HLB-affected and healthy PCRC of three sources (fresh, 6-months and 9-years).

Name	Fold Change (p-value < 0.05 and VIP > 1)		
	fresh PCRC	6-months PCRC	9-years PCRC
**Amino acids**			
L-2-hydroxyglutaric acid	-1.4	1.2	10.0
N-α-acetyllysine	-1.1	1.1	7.9
N2-γ-glutamylglutamine	-1.4	-2.0	4.2
Arginine	2.1	2.9	3.5
threonine	-1.2	1.2	3.3
D-asparagine	1.1	1.3	3.2
Kynurenine	-1.9	-1.5	-2.6
N-acetyl-L-tyrosine	1.6	-1.5	-2.9
N-(L-arginino) succinate	-4.0	-1.2	-5.0
N-acetylglutamic acid	NA	-1.4	14.5
Tryptophan	NA	1.8	3.6
Lysine	NA	1.4	3.3
Ornithine	NA	-1.4	2.3
O-acetyl-L-homoserine	1.2	NA	5.1
Glutamic acid	4.5	NA	4.1
3-hydroxy-5-methyl-L-tyrosine	-1.2	NA	2.8
Pyroglutamic acid	2.6	-7.1	NA
Leucine	NA	NA	9.4
Tyramine	NA	NA	-2.0
Methionine	1.7	NA	-2.4
Aspartic acid	NA	NA	-2.7
N-acetyl-a-neuraminic acid	NA	NA	-3.1
Phenylalanine	NA	NA	-5.6
Pipecolic acid	NA	NA	-7.7
D-β-phenylalanine	9.2	NA	NA
**Polysaccharides**			
Melezitose	1.1	-1.7	8.1
D-Ribose	1.1	1.1	3.9
p-coumaroyl-D-glucose	1.6	1.3	3.7
3'-ketolactose	11.1	1.6	2.5
Cellobiose	3.2	3.3	-1.5
Digalacturonate	6.3	-5.0	-4.8
1-kestose	NA	2.7	3.6
Stachyose	NA	-1.4	3.2
D-galactose	NA	-2.4	-1.3
D-xylose	NA	1.1	-3.6
2-amino-2-deoxy-D-gluconate	NA	1.2	-3.7
N-acetyl-D-glucosamine	NA	1.1	-4.0
Chitobiose	87.4	NA	208.4

D-xylitol	3.2	NA	3.6
Lactose	-1.3	NA	3.5
β-D-Glucosamine	2.1	NA	1.4
1-O-vanilloyl-β-D-glucose	1.3	3.6	NA
Maltotriose	NA	NA	10.8
D-lyxose	NA	NA	8.9
Trehalose	NA	NA	5.4
L-arabinose	NA	NA	3.4
Mannitol	NA	NA	2.6
Methyl β-D-galactoside	NA	NA	2.3
N-acetylmannosamine	NA	NA	-2.1
trans-zeatin-7-β-D-glucoside	NA	-2.5	NA
**Organic acid**			
4-guanidinobutanoic acid	-1.2	-4.2	17.4
4,5-dihydroorotic acid	2.6	-1.5	11.7
Azelaic acid	-6.7	1.9	2.7
9,10-epoxyoctadecenoic acid	1.5	1.3	2.4
Citric acid	1.2	1.6	2.3
Sebacic acid	2.1	1.6	2.0
Succinic acid	1.1	-1.4	-2.2
Traumatic acid	-1.4	2.2	-2.4
Malonate	NA	1.1	150.3
Pantothenic acid	NA	-1.4	66.5
Kojic acid	NA	1.5	4.6
Quinate	2.3	NA	10.4
5-Nitro-2-(3-phenylpropylamino)benzoic acid	4.1	NA	6.9
p-anisic acid	-2.8	NA	-1.6
4-acetamidobenzoic acid	1.1	NA	-2.7
2-pyrocatechuic acid	-1.8	NA	-4.5
Mandelic acid	1.5	6.1	NA
Dodecanedioic acid	-4.3	-2.0	NA
(R)-3-Hydroxybutyric acid	NA	NA	3.3
Diaminopimelic acid	1.2	NA	3.2
10-hydroxydecanoic acid	NA	NA	2.5
12-hydroxydodecanoic acid	NA	NA	-2.2
3-dehydroshikimate	NA	NA	-2.8
Xanthoxic acid	NA	NA	-3.0
Aminoadipic acid	NA	NA	-5.6
Erucic acid	3.3	NA	NA
p-coumaroyl quinic acid	2.2	NA	NA
D-glucarate	-5.6	NA	NA
**Fatty acids**			
Arachidic acid	1.6	-3.8	-1.5

β-glycerophosphoric acid	NA	1.7	13.0
Docosahexaenoic acid	NA	-1.4	5.8
10-nitrolinoleic acid	1.5	NA	-33.3
Linoleic acid	NA	NA	4.2
Pentadecanoic acid	NA	NA	2.5
γ-linolenic acid	NA	NA	-2.3
13-L-hydroperoxylinoleic acid	NA	NA	-10.0
Arachidonic acid	NA	NA	-50.0

[a]NA, No applicable
